# Liver metastases from oropharyngeal squamous cell carcinoma: a rare case report

**DOI:** 10.1093/jscr/rjaf417

**Published:** 2025-07-12

**Authors:** Daniel Solis-Pazmino, Roberta Baldin, Magno Guarçoni, Ricardo Bertinatto, Pedro Maldonado, Andres Cedeno, Rachel Ma, Roberto Pelegrini Coral, Paola Solis-Pazmino

**Affiliations:** Department of Oral and Maxillofacial Surgery, Pontifical Catholic University of Rio Grande do Sul (PUCRS), Avenida Ipiranga, 6681 Partenón, Porto Alegre, RS 90619-900, Brazil; CaTaLiNA- Cancer de Tiroides en Latino América, Quito, Ecuador; Department of General Surgery, Santa Casa de Misericórdia de Porto Alegre, Rua Professor Annes Dias, 295 Centro Histórico, Porto Alegre, RS 90020-090, Brazil; Department of General Surgery, Santa Casa de Misericórdia de Porto Alegre, Rua Professor Annes Dias, 295 Centro Histórico, Porto Alegre, RS 90020-090, Brazil; Department of General Surgery, Santa Casa de Misericórdia de Porto Alegre, Rua Professor Annes Dias, 295 Centro Histórico, Porto Alegre, RS 90020-090, Brazil; Department of General Surgery, Santa Casa de Misericórdia de Porto Alegre, Rua Professor Annes Dias, 295 Centro Histórico, Porto Alegre, RS 90020-090, Brazil; Department of General Surgery, Santa Casa de Misericórdia de Porto Alegre, Rua Professor Annes Dias, 295 Centro Histórico, Porto Alegre, RS 90020-090, Brazil; Surgery Group Los Angeles, 8635 W 3rd St Suite 880W, Los Angeles, CA 90048, United States; Department of General Surgery, Santa Casa de Misericórdia de Porto Alegre, Rua Professor Annes Dias, 295 Centro Histórico, Porto Alegre, RS 90020-090, Brazil; CaTaLiNA- Cancer de Tiroides en Latino América, Quito, Ecuador; Department of General Surgery, Santa Casa de Misericórdia de Porto Alegre, Rua Professor Annes Dias, 295 Centro Histórico, Porto Alegre, RS 90020-090, Brazil; Surgery Group Los Angeles, 8635 W 3rd St Suite 880W, Los Angeles, CA 90048, United States

**Keywords:** oropharyngeal squamous cell carcinoma, liver metastasis, head and neck cancer, case report, incomplete radiotherapy, health system limitations

## Abstract

Oropharyngeal squamous cell carcinoma (OPSCC) commonly metastasizes to the lungs, bones, and distant lymph nodes. Liver metastases from OPSCC are rare and typically occur in advanced stages. We present a 59-year-old male diagnosed with OPSCC, initially treated with surgery followed by adjuvant radiotherapy (RDT). Due to political and governmental limitations, the patient received only six sessions of RDT, below the standard recommended regimen. Three years post-treatment, he presented with abdominal symptoms, and imaging revealed a hepatic lesion. A liver biopsy confirmed metastatic squamous cell carcinoma. Histopathological comparison with the primary tumor supported the diagnosis of hepatic metastasis from OPSCC. This case underscores the aggressive nature of OPSCC and the critical importance of completing multimodal therapy. Interrupted adjuvant treatment may have contributed to early distant spread. While liver metastases from OPSCC are rare, they should be considered in patients presenting with compatible symptoms and a history of incomplete treatment.

## Introduction

Head and neck cancers account for 3% and 4% of the malignancies in the United States and Europe, respectively [1]. The most common histology type is the oropharyngeal squamous cell carcinoma (OPSCC). OPSCC primarily spreads to regional lymph nodes and less frequently metastasizes to distant organs. Distant metastases typically occur in advanced-stage (T3/T4) or recurrent OPSCC, and the common sites include the lungs, bone, and liver. However, liver involvement is relatively rare [2].

Risk factors for distant metastases include the more aggressive HPV-negative OPSCC [3, 4], large primary tumors (T3/T4), extensive nodal involvement (N2/N3) [5, 6], and perineural invasion or extranodal extension [7].

The heterogeneity of cancer tissue is the largest obstacle to treatment and could explain the poor prognosis of advanced cancer patients with distant metastasis [8]. The role of surgery, chemotherapy, and radiotherapy also remain unclear. Palliative care may also be incorporated to manage symptoms and improve the quality of life [9]. We present an unusual presentation of isolated OPSCC liver metastasis following incomplete adjuvant treatment.

## Case presentation

A 60-year-old, male chronic smoker came to the Digestive Surgery Department due to jaundice, associated with choluria, acholia, and weight loss (>10%). He denied fever, nausea, vomiting, or other changes. In 2020, he had a history of p16 positive OPSCC with pathological TNM staging as pT1pN3M0. The tumor was treated 2 years prior with resection with oncological-free margins, six sessions of adjuvant radiotherapy, and cisplatin chemotherapy.

**Figure 1:**
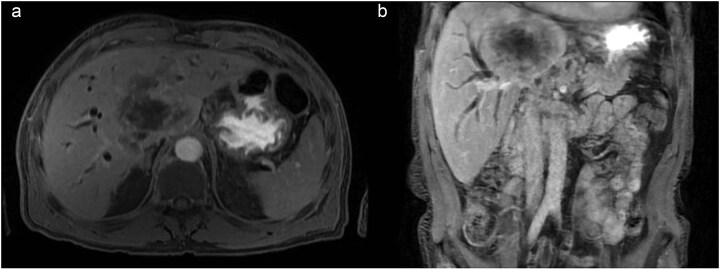
(a) Axial contrast enhanced CT scan shows a central infiltrative lesion in the hepatic parenchyma, affecting segments I, IV, and VIII, measuring 11.0 × 8.8 cm. (b) Coronal contrast enhanced CT scan the mass causing obstruction of the bile duct at the confluence of the hepatic ducts and involvement of segmental ducts of both lobes.

During admission, the blood tests reported abnormal liver enzyme levels: total bilirubin of 17.9 mg/dl, direct bilirubin of 13.9 mg/dl, aspartate aminotransferase (AST) of 108 U/L, alanine transaminase (ALT) of 118 U/L, gamma-glutamyl transferase of 1347 IU/L, and alkaline phosphatase of 588 IU/L. The blood tests also reported abnormal coagulation levels: INR of 2.04, activated partial thromboplastin time of 41.5 s. An abdominal CT scan revealed a central infiltrative lesion in the hepatic parenchyma, affecting segments I, IV, and VIII, measuring ~11.0 × 8.8 cm (Fig. 1a). There was an obstruction of the bile duct at the confluence of the hepatic ducts and involvement of segmental ducts of both lobes with dilation of the intrahepatic bile ducts (Fig. 1b). The lesion was in contact with the middle hepatic vein, the distal segment of the portal vein trunk, the right and left portal branches, and the retro hepatic inferior vena cava. Thus, the diagnosis of neoplasia was suspected, and both hepatocellular carcinoma and cholangiocarcinoma were considered.

**Figure 2:**
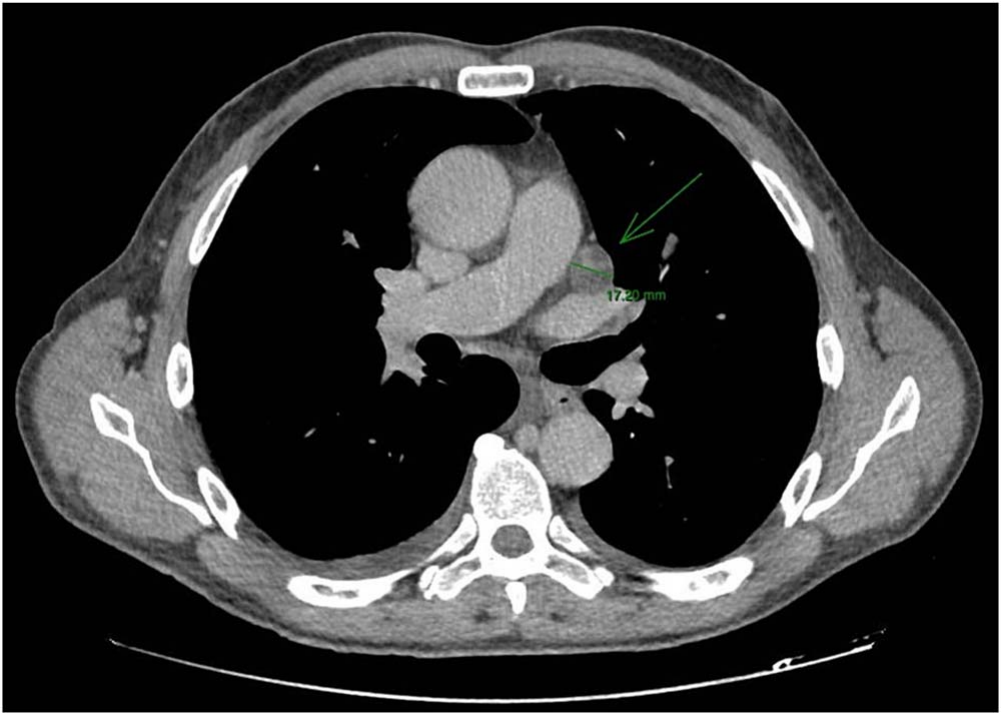
CT staging of the chest and pelvis showed the presence of subaortic, peri-esophageal, and hilar necrotic lymph node enlargement on the left, suggestive of secondary implants.

**Figure 3:**
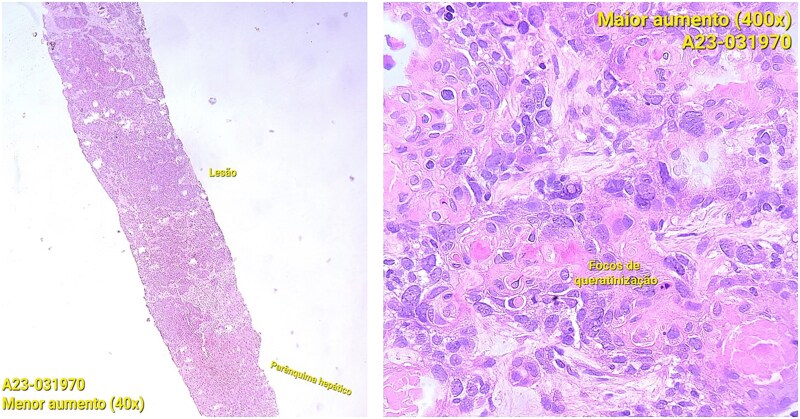
Photomicrographs of orophyarngeal biopsy showing squamous cell carcinoma: Islands of tumor cells along with chronic inflammatory infiltrate (hematoxylin and eosin staining).

**Figure 4:**
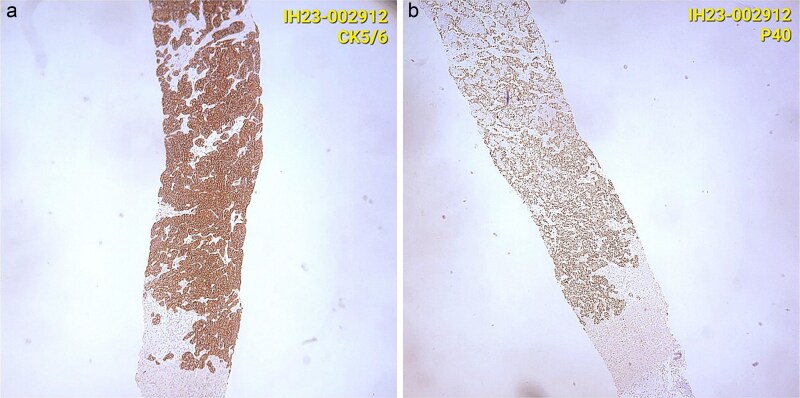
(a) Immunohistochemically, the tumor cells were cytokeratin 5/6(+). (b) Immunohistochemically, the tumor cells were p40 (+).

CT staging of the chest and pelvis showed the presence of subaortic, peri-esophageal, and hilar necrotic lymph node enlargement on the left, suggestive of secondary implants (Fig. 2). Then, a biopsy of the largest liver lesion showed a metastasis of OPSCC. Individual cells were large and pleomorphic with moderate amount of eosinophilic cytoplasm and high nuclear-cytoplasmic ratio. Nuclei were large, pleomorphic, and hyperchromatic with prominent nuclei (Fig. 3). Immunohistochemically, the tumor cells were cytokeratin 5/6(+) (Fig. 4a) and p40 (+) (Fig. 4b). The liver biopsy was compared with the primary OPSCC histological specimen. Morphological features and immunohistochemical profiles were consistent with metastatic oropharyngeal squamous cell carcinoma.

After a tumor board meeting, we declined the surgical option due to the extensive liver metastasis. The patient was given palliative care and died six months after the metastatic diagnosis.

## Discussion

We presented a patient with liver metastasis from an OPSCC. Liver metastasis from head and neck cancer (HNC) are rare (0.7–0.9%) [10]. It has limited options for treatment and poor prognosis, with an overall survival of <6 months [11].

Detection of liver distant metastasis (LDM) from OPSCC is rare. Most studies agree that distant metastases most commonly present 2 years after the diagnosis of the primary tumor [12]. The most common cause is an incomplete course of adjuvant radiotherapy, representing a significant deviation from standard management protocols for OPSCC. Multiple studies have demonstrated that the completion of planned adjuvant radiotherapy is associated with improved locoregional control and overall survival. For instance, in a review paper, Gonzalez Ferreira *et al.* [13], reported that radiotherapy interruptions or prolonged overall treatment time had deleterious detrimental effects on both local control and survival in patients with HNC. Similarly, a prospective observational analysis by Alapati *et al*. [14] emphasized that HNSCC patients treated with radiation at academic medical centers reported significantly greater phys-Quality of Life (QoL) in their first-year post-treatment compared to those treated at community medical centers. In contrast to these findings, our patient was unable to complete the standard regimen due to external political and governmental limitations. This highlights the broader issue of disparities in access to healthcare, which can undermine the effectiveness of established treatment protocols and may contribute to earlier recurrence or distant metastasis, as observed in our case.

There is controversial regarding the treatment in LDM from OPSCC. The role of surgery, chemotherapy, radiotherapy, and palliative care remain unclear. Marcy *et al.* [10]. reported that none of the LDM patients underwent surgery (17 treated with chemotherapy and 7 with supportive care) due to poor prognosis. Palliative care plays a crucial role in managing symptoms and improving the quality of life for patients with liver metastases [12, 15]. Multidisciplinary care involving oncologists, hepatologists, pain specialists, and palliative care teams is essential to address the complex needs of these patients.

The prognosis of LDM from HNSCC is poor. In a retrospective study using the Surveillance, Epidemiology, and End Results database, Cai *et al.* [8] showed that older, uninsured male patients with T3/T4 stage and N1-3 had poor prognosis, with a median overall survival of 4.0 months. Our patient had a pT1pN3M0 tumor, and he died six-months after the LDM detection. Kjems *et al.* [9], in a retrospective study of 7300 patients, compared distant metastasis at the time of primary diagnosis vs. >3 months LDM. They found that p16 (+) tumors had lower number of LDMs vs. p16 (−) tumors (6% vs. 10%). Also, LDMs from p16 (+) tumors had superior survival. Although our patient had P16 positive, he died earlier.

A major limitation in this case was the incomplete adjuvant radiotherapy, which consisted of only six sessions. This deviation from standard treatment protocols was not due to clinical or patient-related factors, but rather due to external political and governmental limitations that restricted access to radiotherapy services. This interruption may have influenced disease progression and highlights the impact of systemic barriers on oncologic outcomes.

## Conclusion

This case illustrates a rare instance of liver metastasis from oropharyngeal squamous cell carcinoma, emphasizing the potential for atypical metastatic patterns, particularly in patients who do not complete standard treatment protocols. The early appearance of distant metastases in our patient may have been influenced by the interruption of adjuvant radiotherapy due to external political and governmental barriers. This highlights the critical importance of ensuring access to uninterrupted, guideline-based cancer care and underscores the need for clinicians to maintain a high index of suspicion for uncommon metastatic sites in OPSCC follow-up.
